# 2-(4-Bromo­phen­yl)quinoxaline

**DOI:** 10.1107/S160053681001723X

**Published:** 2010-05-19

**Authors:** Zhi-jian Wang, Wei-min Jia, Hong-guo Yao, Hong Qiu, Wei Wang

**Affiliations:** a School of Perfume and Aroma Technology, Shanghai Institute of Technology, Shanghai 200235, People’s Republic of China; b School of Chemical Engineering, University of Science and Technology, Liaoning Anshan 114051, People’s Republic of China

## Abstract

In the title compound, C_14_H_9_BrN_2_, the benzene and quinoxaline rings are almost coplanar [r.m.s. deviation = 0.0285 (3) Å and dihedral angle = 2.1 (2)°].

## Related literature

For the synthesis of quinoxaline derivatives, see: Raw *et al.* (2003 ▶); Bhosale *et al.* (2005 ▶). For their applications, see: Brock *et al.* (1999 ▶); Seitz *et al.* (2002 ▶); He *et al.* (2003 ▶). For typical bond lengths in a related structure, see: Rong *et al.* (2006 ▶).
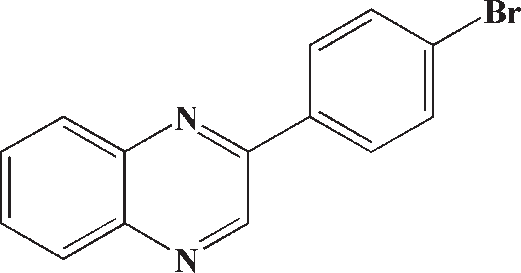


## Experimental

### 

#### Crystal data

C_14_H_9_BrN_2_
*M* *_r_* = 285.14
Monoclinic, 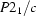

*a* = 13.959 (3) Å
*b* = 5.9031 (12) Å
*c* = 14.497 (3) Å
β = 109.53 (3)°
*V* = 1125.9 (4) Å^3^
*Z* = 4
Mo *K*α radiation
μ = 3.63 mm^−1^
*T* = 153 K
0.20 × 0.18 × 0.10 mm

#### Data collection

Rigaku MM-OO7/Saturn 70 CCD area-detector diffractometer
Absorption correction: multi-scan (*REQAB*; Jacobson, 1998 ▶) *T*_min_ = 0.531, *T*_max_ = 0.713
8910 measured reflections
2683 independent reflections
1763 reflections with *I* > 2σ(*I*)
*R*_int_ = 0.052

#### Refinement

*R*[*F* ^2^ > 2σ(*F* ^2^)] = 0.032
*wR*(*F* ^2^) = 0.075
*S* = 0.96
2683 reflections
155 parameters
H-atom parameters constrained
Δρ_max_ = 0.76 e Å^−3^
Δρ_min_ = −0.69 e Å^−3^

### 

Data collection: *CrystalClear* (Rigaku/MSC, 2005 ▶); cell refinement: *CrystalClear*; data reduction: *CrystalClear*; program(s) used to solve structure: *SHELXS97* (Sheldrick, 2008 ▶); program(s) used to refine structure: *SHELXL97* (Sheldrick, 2008 ▶); molecular graphics: *SHELXTL* (Sheldrick, 2008 ▶); software used to prepare material for publication: *SHELXTL*.

## Supplementary Material

Crystal structure: contains datablocks global, I. DOI: 10.1107/S160053681001723X/zs2039sup1.cif

Structure factors: contains datablocks I. DOI: 10.1107/S160053681001723X/zs2039Isup2.hkl

Additional supplementary materials:  crystallographic information; 3D view; checkCIF report

## Figures and Tables

**Fig. 1.**
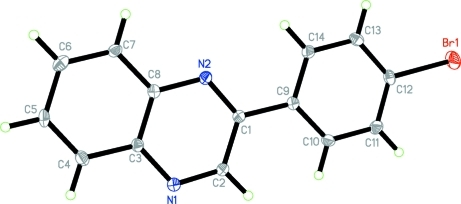
Molecular configuration of the molecule of (I) showing the atom-labelling scheme. Displacement ellipsoids are drawn at the 35% probability level.

**Fig. 2.**
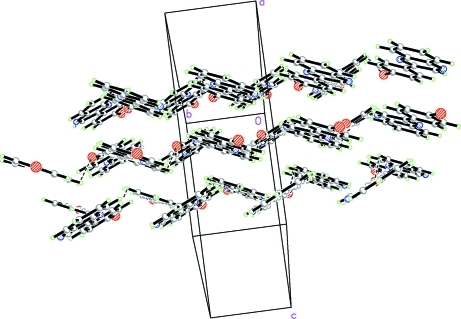
The crystal packing of (I), viewed down the c axis.

## supplementary crystallographic information

### Comment

Quinoxaline derivatives are an important class of nitrogen containing heterocycles, finding use as intermediates in organic synthesis and in addition have been reported as having applications as anticancer, antiviral, and antibacterial agents (Seitz *et al.*, 2002; He *et al.*, 2003) and dyes (Brock *et al.*, 1999). In recent years, many syntheses of quinoxaline derivatives have been reported (Raw *et al.*, 2003; Bhosale *et al.*, 2005). The title compound C_14_H_9_BrN_2_ (I) is one of such quinoxaline derivates which we have synthesized and now report its crystal structure.

The molecular structure of title compound is as shown in Fig.1. The bond lengths and angles are usual for this type of compound (Rong *et al.*, 2006). The dihedral angle between the benzene ring and quinoxaline ring is 2.1 (2)°, which means that the benzene ring and the quinoxaline ring are approximately coplanar with a r.m.s deviation of 0.0285 (3) Å, the Br atom lying in the plane of the substituent benzene ring [r.m.s deviation, 0.0271 (3) Å]. The crystal packing (Fig. 2) is stabilized by van der Waals forces.

### Experimental

A suspension of hydrated 2-(4-bromophenyl)-2-oxoacetaldehyde (2.0 mmol) and benzene-1,2-diamine (3.0 mmol) in ethanol (5 ml) was stirred at room temperature with the reaction progress monitored *via* TLC. The resulting precipitate was filtered off, washed with cold ethanol, dried and purified to give the title compound as a light yellow solid (92.5% yield: m.p. 418 K). Crystals suitable for single-crystal X-ray analysis were grown by slow evaporation of a solution in chloroform-ethanol (1:1).

### Refinement

All H atoms were positioned geometrically and refined as riding with C—H = 0.95 Å and *U*_iso_(H) set equal to 1.2*U*_eq_(carrier atom).

### Crystal data

**Table:**

C_14_H_9_BrN_2_	*F*(000) = 568
*M**_r_* = 285.14	*D*_x_ = 1.682 Mg m^−^^3^
Monoclinic, *P*2_1_/*c*	Melting point: 418 K
Hall symbol: -P 2ybc	Mo *K*α radiation, λ = 0.71073 Å
*a* = 13.959 (3) Å	Cell parameters from 3221 reflections
*b* = 5.9031 (12) Å	θ = 2.9–27.9°
*c* = 14.497 (3) Å	µ = 3.63 mm^−^^1^
β = 109.53 (3)°	*T* = 153 K
*V* = 1125.9 (4) Å^3^	Prism, colorless
*Z* = 4	0.20 × 0.18 × 0.10 mm

### Data collection

**Table:**

Rigaku Model name? CCD area-detector diffractometer	2683 independent reflections
Radiation source: rotating anode	1763 reflections with *I* > 2σ(*I*)
multilayer	*R*_int_ = 0.052
Detector resolution: 7.31 pixels mm^-1^	θ_max_ = 27.9°, θ_min_ = 2.9°
φ and ω scans	*h* = −18→11
Absorption correction: multi-scan (*REQAB*; Jacobson, 1998)	*k* = −7→7
*T*_min_ = 0.531, *T*_max_ = 0.713	*l* = −19→19
8910 measured reflections	

### Refinement

**Table:**

Refinement on *F*^2^	Secondary atom site location: difference Fourier map
Least-squares matrix: full	Hydrogen site location: inferred from neighbouring sites
*R*[*F*^2^ > 2σ(*F*^2^)] = 0.032	H-atom parameters constrained
*wR*(*F*^2^) = 0.075	*w* = 1/[σ^2^(*F*_o_^2^) + (0.0337*P*)^2^] where *P* = (*F*_o_^2^ + 2*F*_c_^2^)/3
*S* = 0.96	(Δ/σ)_max_ = 0.001
2683 reflections	Δρ_max_ = 0.76 e Å^−^^3^
155 parameters	Δρ_min_ = −0.69 e Å^−^^3^
0 restraints	Extinction correction: *SHELXL97* (Sheldrick, 2008), Fc^*^=kFc[1+0.001xFc^2^λ^3^/sin(2θ)]^-1/4^
Primary atom site location: structure-invariant direct methods	Extinction coefficient: 0.0540 (17)

### Special details

**Table:**

Geometry. All esds (except the esd in the dihedral angle between two l.s. planes) are estimated using the full covariance matrix. The cell esds are taken into account individually in the estimation of esds in distances, angles and torsion angles; correlations between esds in cell parameters are only used when they are defined by crystal symmetry. An approximate (isotropic) treatment of cell esds is used for estimating esds involving l.s. planes.
Refinement. Refinement of *F*^2^ against ALL reflections. The weighted *R*-factor wR and goodness of fit *S* are based on *F*^2^, conventional *R*-factors *R* are based on *F*, with *F* set to zero for negative *F*^2^. The threshold expression of *F*^2^ > σ(*F*^2^) is used only for calculating *R*-factors(gt) etc. and is not relevant to the choice of reflections for refinement. *R*-factors based on *F*^2^ are statistically about twice as large as those based on *F*, and *R*- factors based on ALL data will be even larger.

### Fractional atomic coordinates and isotropic or equivalent isotropic displacement parameters (Å^2^)

**Table:**

	*x*	*y*	*z*	*U*_iso_*/*U*_eq_	
Br1	0.460263 (18)	1.10538 (4)	0.33474 (2)	0.03213 (13)	
N1	−0.12149 (15)	0.6560 (3)	0.02060 (15)	0.0218 (5)	
N2	−0.05847 (15)	1.0700 (3)	0.12055 (14)	0.0169 (4)	
C1	0.00712 (17)	0.9095 (4)	0.11986 (16)	0.0150 (5)	
C2	−0.02653 (17)	0.7012 (4)	0.07034 (17)	0.0210 (6)	
H2	0.0229	0.5882	0.0736	0.025*	
C3	−0.19054 (16)	0.8203 (4)	0.02054 (16)	0.0160 (5)	
C4	−0.29514 (17)	0.7808 (4)	−0.02863 (17)	0.0204 (6)	
H4	−0.3168	0.6436	−0.0636	0.024*	
C5	−0.36496 (18)	0.9398 (4)	−0.02576 (17)	0.0213 (6)	
H5	−0.4352	0.9126	−0.0588	0.026*	
C6	−0.33379 (19)	1.1438 (4)	0.02573 (18)	0.0229 (6)	
H6	−0.3833	1.2523	0.0279	0.028*	
C7	−0.23291 (17)	1.1877 (4)	0.07274 (17)	0.0176 (5)	
H7	−0.2126	1.3275	0.1060	0.021*	
C8	−0.15888 (16)	1.0252 (4)	0.07190 (15)	0.0150 (5)	
C9	0.11683 (17)	0.9532 (4)	0.17107 (16)	0.0157 (5)	
C10	0.19145 (17)	0.7987 (4)	0.16884 (17)	0.0186 (5)	
H10	0.1721	0.6609	0.1337	0.022*	
C11	0.29343 (17)	0.8427 (4)	0.21702 (18)	0.0206 (6)	
H11	0.3437	0.7360	0.2152	0.025*	
C12	0.32117 (17)	1.0434 (4)	0.26773 (17)	0.0186 (5)	
C13	0.24914 (17)	1.2017 (4)	0.27042 (17)	0.0196 (5)	
H13	0.2692	1.3406	0.3045	0.023*	
C14	0.14782 (18)	1.1554 (4)	0.22297 (16)	0.0186 (5)	
H14	0.0980	1.2627	0.2255	0.022*	

### Atomic displacement parameters (Å^2^)

**Table:**

	*U*^11^	*U*^22^	*U*^33^	*U*^12^	*U*^13^	*U*^23^
Br1	0.01429 (15)	0.0399 (2)	0.03931 (19)	−0.00711 (11)	0.00519 (12)	−0.00733 (13)
N1	0.0176 (11)	0.0191 (10)	0.0248 (11)	0.0017 (8)	0.0021 (9)	−0.0045 (9)
N2	0.0157 (10)	0.0175 (10)	0.0176 (9)	0.0015 (8)	0.0054 (9)	−0.0003 (8)
C1	0.0153 (11)	0.0153 (12)	0.0148 (11)	0.0002 (9)	0.0058 (10)	0.0002 (9)
C2	0.0158 (12)	0.0198 (12)	0.0246 (13)	0.0035 (10)	0.0029 (11)	−0.0052 (11)
C3	0.0148 (12)	0.0172 (11)	0.0147 (11)	0.0002 (9)	0.0031 (10)	0.0018 (9)
C4	0.0188 (13)	0.0194 (12)	0.0193 (11)	−0.0026 (10)	0.0015 (11)	0.0002 (10)
C5	0.0115 (12)	0.0273 (14)	0.0216 (12)	0.0004 (9)	0.0009 (10)	0.0039 (10)
C6	0.0248 (14)	0.0213 (13)	0.0234 (13)	0.0085 (11)	0.0090 (12)	0.0048 (10)
C7	0.0188 (12)	0.0135 (11)	0.0209 (12)	0.0022 (10)	0.0070 (11)	0.0018 (10)
C8	0.0149 (12)	0.0165 (12)	0.0141 (11)	−0.0005 (9)	0.0053 (10)	0.0022 (9)
C9	0.0149 (12)	0.0169 (12)	0.0151 (11)	−0.0009 (9)	0.0048 (10)	0.0013 (9)
C10	0.0173 (12)	0.0158 (11)	0.0227 (12)	−0.0020 (10)	0.0068 (10)	−0.0043 (10)
C11	0.0156 (12)	0.0210 (13)	0.0268 (13)	0.0021 (10)	0.0090 (11)	−0.0011 (10)
C12	0.0124 (11)	0.0229 (13)	0.0198 (12)	−0.0047 (9)	0.0045 (10)	0.0007 (10)
C13	0.0212 (13)	0.0171 (11)	0.0203 (12)	−0.0051 (10)	0.0068 (11)	−0.0018 (10)
C14	0.0182 (12)	0.0200 (13)	0.0172 (11)	0.0029 (10)	0.0055 (11)	−0.0018 (10)

### Geometric parameters (Å, °)

**Table:**

Br1—C12	1.894 (2)	C6—C7	1.369 (3)
N1—C2	1.307 (3)	C6—H6	0.9500
N1—C3	1.367 (3)	C7—C8	1.413 (3)
N2—C1	1.320 (3)	C7—H7	0.9500
N2—C8	1.368 (3)	C9—C10	1.393 (3)
C1—C2	1.422 (3)	C9—C14	1.400 (3)
C1—C9	1.484 (3)	C10—C11	1.385 (3)
C2—H2	0.9500	C10—H10	0.9500
C3—C8	1.411 (3)	C11—C12	1.379 (3)
C3—C4	1.414 (3)	C11—H11	0.9500
C4—C5	1.364 (3)	C12—C13	1.383 (3)
C4—H4	0.9500	C13—C14	1.378 (3)
C5—C6	1.407 (3)	C13—H13	0.9500
C5—H5	0.9500	C14—H14	0.9500
			
C2—N1—C3	116.11 (19)	C8—C7—H7	120.0
C1—N2—C8	116.82 (18)	N2—C8—C3	121.6 (2)
N2—C1—C2	120.8 (2)	N2—C8—C7	119.4 (2)
N2—C1—C9	118.30 (19)	C3—C8—C7	119.0 (2)
C2—C1—C9	120.9 (2)	C10—C9—C14	118.1 (2)
N1—C2—C1	123.9 (2)	C10—C9—C1	121.96 (19)
N1—C2—H2	118.0	C14—C9—C1	119.9 (2)
C1—C2—H2	118.0	C11—C10—C9	121.1 (2)
N1—C3—C8	120.74 (19)	C11—C10—H10	119.5
N1—C3—C4	119.5 (2)	C9—C10—H10	119.5
C8—C3—C4	119.7 (2)	C12—C11—C10	119.2 (2)
C5—C4—C3	119.9 (2)	C12—C11—H11	120.4
C5—C4—H4	120.0	C10—C11—H11	120.4
C3—C4—H4	120.0	C11—C12—C13	121.2 (2)
C4—C5—C6	120.5 (2)	C11—C12—Br1	119.76 (18)
C4—C5—H5	119.7	C13—C12—Br1	119.03 (17)
C6—C5—H5	119.7	C14—C13—C12	119.1 (2)
C7—C6—C5	120.7 (2)	C14—C13—H13	120.4
C7—C6—H6	119.7	C12—C13—H13	120.4
C5—C6—H6	119.7	C13—C14—C9	121.3 (2)
C6—C7—C8	120.1 (2)	C13—C14—H14	119.4
C6—C7—H7	120.0	C9—C14—H14	119.4
			
C8—N2—C1—C2	0.1 (3)	C4—C3—C8—C7	−0.2 (3)
C8—N2—C1—C9	179.40 (19)	C6—C7—C8—N2	−179.0 (2)
C3—N1—C2—C1	−2.1 (3)	C6—C7—C8—C3	1.2 (3)
N2—C1—C2—N1	2.2 (4)	N2—C1—C9—C10	−176.0 (2)
C9—C1—C2—N1	−177.1 (2)	C2—C1—C9—C10	3.3 (4)
C2—N1—C3—C8	0.0 (3)	N2—C1—C9—C14	3.5 (3)
C2—N1—C3—C4	−177.7 (2)	C2—C1—C9—C14	−177.2 (2)
N1—C3—C4—C5	177.3 (2)	C14—C9—C10—C11	0.4 (4)
C8—C3—C4—C5	−0.4 (3)	C1—C9—C10—C11	179.9 (2)
C3—C4—C5—C6	0.1 (4)	C9—C10—C11—C12	−0.2 (4)
C4—C5—C6—C7	0.8 (4)	C10—C11—C12—C13	−0.6 (4)
C5—C6—C7—C8	−1.5 (4)	C10—C11—C12—Br1	179.59 (19)
C1—N2—C8—C3	−2.2 (3)	C11—C12—C13—C14	1.2 (4)
C1—N2—C8—C7	178.0 (2)	Br1—C12—C13—C14	−179.02 (17)
N1—C3—C8—N2	2.2 (3)	C12—C13—C14—C9	−1.0 (4)
C4—C3—C8—N2	179.9 (2)	C10—C9—C14—C13	0.2 (4)
N1—C3—C8—C7	−177.9 (2)	C1—C9—C14—C13	−179.4 (2)
